# Decreased H19, GAS5, and linc0597 Expression and Association Analysis of Related Gene Polymorphisms in Rheumatoid Arthritis

**DOI:** 10.3390/biom10010055

**Published:** 2019-12-29

**Authors:** Jun Wu, Tian-Ping Zhang, Yu-Lan Zhao, Bao-Zhu Li, Rui-Xue Leng, Hai-Feng Pan, Dong-Qing Ye

**Affiliations:** 1 Department of Epidemiology and Biostatistics, School of Public Health, Anhui Medical University, 81 Meishan Road, Hefei 230032, China; wj1309230394@126.com (J.W.); 15715511358@163.com (T.-P.Z.); dqjkzhao@126.com (Y.-L.Z.); libaozhu@ahmu.edu.cn (B.-Z.L.); lengruixue@ahmu.edu.cn (R.-X.L.); 2 Anhui Province Key Laboratory of Major Autoimmune Diseases, Anhui Medical University, 81 Meishan Road, Hefei 230032, China

**Keywords:** Rheumatoid arthritis, Long noncoding RNAs, Single nucleotide polymorphisms

## Abstract

Long noncoding RNAs (lncRNAs) widely participate in human diseases by regulating gene transcription, modulating protein function, or acting as ceRNAs. Yet, their roles in rheumatoid arthritis (RA) remain obscure. In this study, the expression of three lncRNAs (H19, GAS5, and linc0597) in peripheral blood mononuclear cells (PBMCs) were detected in 77 RA patients and 78 controls using quantitative real-time reverse transcription polymerase chain reaction (qRT-PCR). The association of lncRNAs related gene polymorphisms with RA were evaluated in 828 RA patients and 780 controls using TaqMan single nucleotide polymorphism (SNP) genotyping assays. We observed that the expression levels of H19, GAS5 and linc0597 were down-regulated in PBMCs of RA patients, of which GAS5 level decreased in patients with hypocomplementemia, and negatively correlated with C-reactive protein (CRP) level in RA patients. Moreover, we highlighted two related potential functional SNPs, GAS5 rs6790 and linc0597 rs2680700 for associations with RA susceptibility. The precise roles of these lncRNAs in mechanism of RA remain to be further explored.

## 1. Introduction

Rheumatoid arthritis (RA) is a chronic autoimmune disease characterized by autoantibodies, systemic inflammation, persistent synovitis, and irreversible joint cartilage and bone destruction [1]. Some extra-articular symptoms, such as pleuritis and rheumatoid vasculitis, can cause complications and poor prognosis of RA [1]. To date, RA affects approximately 1% of the global population [2]. However, the certain aetiology and the exact pathogenesis of RA remains obscure. Recently, clinical trials and epidemiological data have indicated that the interaction of environmental factors, genetic factors, autoimmunity, endocrine system, and infection contributes to the initiation and progression of RA [1,3,4]. Among them, the dysregulation of innate and adaptive immune response, including aberrant production of immune cells and imbalance of cytokines, were widely demonstrated to participate in RA [5,6]. Abundant genetic variants were broadly confirmed to play major roles in RA [1]. Recently, emerging evidence has revealed that a subset of noncoding RNAs (ncRNAs) also play pivotal roles in RA [7].

It has been shown that merely 1–2% of human genome encode amino acids and 80% of phenotype-related loci map to noncoding regions across the human genome [8,9]. NcRNAs take up a significant portion of non-coding regions [10]. Currently, the majority of research attention in thousands of ncRNAs coded by mammalian genome has shown that ncRNAs may play pivotal roles in diverse biological functions of RA [7,11]. For instance, miRNAs may promote numerous immune processes, and their dysregulation and implications in RA are well accepted [7], such as miR-155 targeting TAB2 to activation of inflammatory responses [12], miR-16 targeting 3′-untranslated region (UTR) of TNF-α to be a signature of disease activity [13], miR-124a acting as a disease biomarker and a potential target for treatment [14], etc. Importantly, research on another type of ncRNAs named lncRNAs in RA is on the rise. LncRNAs, which were initially considered to be a kind of nonfunctional RNAs, were actually capable of manipulating gene expression, such as gene transcription, RNA splicing, chromosome remodeling and protein transport [11,15]. Besides, lncRNAs have the ability to associate with RNA molecules to regulate their translation such as mRNA, miRNA, and interact with proteins to modulate their functions [16]. For instance, linc-p21 was activated by P53 for cell apoptosis by binding to ribonucleoprotein-K [17]. miR-21 and GAS5 may inhibit each other’s expression due to negative correlation between the two lncRNAs [18]. Moreover, lncRNAs as important ceRNAs to impact the distribution of miRNAs on their targets also have received extensive attention [19].

Increasing evidence has supported significant regulatory roles of lncRNAs in inflammatory responses, such as regulating the differentiation and activation of immune cells [15]. In this term, several lncRNAs exhibited aberrant expression profiles in inflamed joint tissues of RA. Hotair is one of the earliest identified lncRNAs with abnormal expression in RA patients, it may interact with polycomb repressive complex 2 to regulate chromatin status [20]. Compared with normal tissues, Hotair overexpressed in RA cartilages [21]. Stuhlmüller et al. found that H19 expression was up-regulated in RA synovial tissue and might be induced by cytokines in synovial fibroblasts [22]. Wu et al. showed that linc0597 (BZRAP1-AS1) decreased in peripheral blood mononuclear cells (PBMCs) of patients with RA [23]. GAS5 originally discovered as a highly expressed ncRNA in growth-retarded cells was another aberrantly expressed lncRNA in RA [24]. Mayama et al. demonstrated that GAS5 expression decreased in B cells and CD4 + T-cells of RA patients [25]. Conversely, Moharamoghli et al. reported that GAS5 expression was up-regulated in T-cells of RA patients [26]. Thus, compelling evidence showing the relevance of lncRNAs with RA is emerging, and in-depth research on the role of lncRNAs in RA is an urgent issue.

To further explore the implications of lncRNAs in RA, this study based on the available evidence hypothesized that H19, linc0597 and GAS5 may be involved in the pathogenesis of RA. A two-stage study of lncRNAs in RA was established. We aimed to investigate the three lncRNAs expression levels in PBMCs of RA patients, as well as explore the associations of lncRNAs expression and their gene single nucleotide polymorphisms (SNPs) with RA susceptibility.

## 2. Materials and Methods

### 2.1. Patients and Controls

During the years 2016–2017, 77 RA patients and 78 controls were enrolled to investigate the expression of H19, GAS5 and linc0597 in PBMCs, 828 RA patients and 780 controls were recruited to explore the association of gene polymorphisms with disease susceptibility. RA patients diagnosed according to 1987 revised American College of Rheumatology (ACR) diagnostic criteria [27] were recruited from Anhui Provincial Hospital and the First Affiliated Hospital of Anhui Medical University. Controls were selected from health blood donors, the physical examination center of the First Affiliated Hospital of Anhui Medical University, and the Second Affiliated Hospital of Anhui Medical University. RA disease severity was evaluated with DAS28-ESR [28], available at http://www.das-score.nl. Exclusion criteria of patients were as follows: (1) Patients suspected of alcohol or drug abuse; (2) Patients complicated with other autoimmune diseases, cancers, systemic infectious diseases, severe heart liver and renal failure; (3) Patients with serious acute infections within one month before admission. Inclusion criteria of controls were as follows: (1) The general Han population; (2) With no history of autoimmune diseases or chronic diseases, including their families; (3) Without any other inflammatory rheumatologic conditions or major diseases; (4) Have not taken hormones or immunosuppressant drugs within one month before admission. Demographic, disease activity and laboratory parameter were collected from questionnaires and medical records. Study was approved by the Medical Ethics Committee of Anhui Medical University (Anhui Medical University 20180078), was conducted according to Declaration of Helsinki principles, and informed consent was obtained from each subject.

### 2.2. Quantitative Real-Time Reverse Transcription Polymerase Chain Reaction

After obtaining informed consent, we collected 5 mL EDTA-anticoagulated blood sample from study subject. PBMCs were freshly isolated from peripheral blood within 4 h and stored at −80 °C. Total RNAs were extracted from PBMCs using TRIzol reagent. In addition, the concentrations and purity of these total RNA samples were measured by NanoDrop™2000 spectrophotometer (Thermo Scientific, Waltham, MA, USA). All of the qualified total RNAs were reverse-transcribed into cDNA based on instructions of the Prime Script TM RT reagent Kit (Takara Bio Inc, Shiga Prefecture, Japan), and stored at −80 °C for further detection.

The qRT-PCR was carried out in duplicate in an optical 96-well plate with a MyCyclerTM Thermal Cycler system by using ABI ViiA™ 7 Real-Time PCR System (Applied Biosystems, Foster City, CA, USA). The qRT-PCR primer sequences are listed in Table 1. Housekeeping gene β-actin was used as internal control. According to specifications of SYBR Green (SYBR^®^ Premix Ex Taq™ II, Takara Bio Inc, Shiga Prefecture, Japan), 0.4 μL forward primer and 0.4 μL reverse primer (10 μM) were applied into qRT-PCR reaction system. Thermal cycling conditions were as follows: 95 °C for 1 min, followed by 42 cycles at 95 °C for 10 s, 60 °C for 30 s and 72 °C for 1 min. 2−∆∆Ct method normalized to endogenous control was used for calculating the relative expression levels of lncRNAs.

### 2.3. Genotyping

The selection of SNPs was based on the available references, and in combine with expression Quantitative Trait Locus (e-QTLs) data of the three lncRNAs from the Genotype Tissue Expression (GTEx) database (https://gtexportal.org/home/). Then, location of genetic information for selected SNPs was verified by data bases of National Center for Biotechnology Information (NCBI) and Ensemble. The minor allele frequencies of all SNPs must be more than 0.05 in a Chinese Han population. In addition, we preferred to select SNPs located in their flanking 2000 bp regions, as well as located in important functional positions, such as 3′UTR, 5′UTR, nonsynonymous mutations. and frameshift mutations. Finally, fourteen SNPs (rs2067051 and rs2075745 for H19; rs6790, rs16847206, and rs6692753 for GAS5; rs2070107, rs2877877, rs2632516, rs2285991, rs8071916, rs12601867, rs13414, rs2680700, and rs4372750 for linc0597) were enrolled into the analysis.

According to the instructions of the Flexi Gene-DNA Kit (Qiagen, Valencia, CA, USA), genomic DNAs were extracted from peripheral blood leukocytes. All SNPs were genotyped by an EP1 platform (Fluidigm, South San Francisco, CA, USA) using TaqMan SNP genotyping assays in accordance with manufacturer’s instructions.

### 2.4. Statistical Analysis

Data were visualized and analyzed with SPSS 23.0 and GraphPad Prism 5.0 (GraphPad Software, La Jolla, CA, USA). Data based on their types were expressed as frequency, percentage, mean ± standard deviation (SD) or median (interquartile range, IQR) respectively. The differences of each lncRNA expression within two groups were analyzed by nonparametric Mann-Whitney U test. Correlation analysis employed the Spearman’s rank correlation coefficient test. The distribution of genotypic and allelic frequencies within each group was evaluated with chi-square test (*χ*^2^) or fisher’s exact test, odds ratio (OR) and 95% confidence interval (CI) were determined by logistic regression analysis. Besides, dominant model and recessive model were included in the analysis. A two-tailed *p* value ≤ 0.05 was considered statistically significant.

## 3. Results

This two-stage case-control study was conducted in a Han Chinese population. Seventy-seven RA patients and 78 controls were recruited to investigate expression of H19, GAS5 and linc0597 in PBMCs in stage one. Eight-hundred twenty-eight RA patients and 780 controls were enrolled to detect gene polymorphisms of differentially expressed lncRNAs in stage two. There were no differences in gender and age distribution between patients and controls at both two stages (all *p* > 0.05). All SNPs genotyped successfully were in accordance with Hardy-Weinberg equilibrium (all *p* >0.05) (Tables S1 and S2).

### 3.1. LncRNAs Expression in PBMCs of Patients with RA.

**Figure 1:**
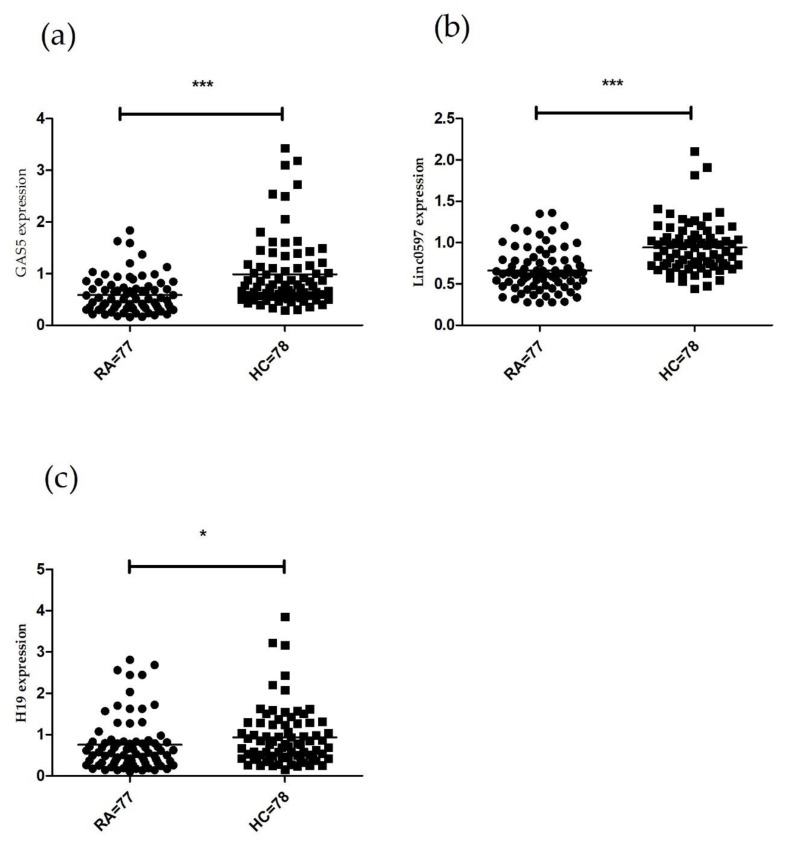
Comparison of GAS5, linc0597 and H19 expression levels in PBMCs between RA patients and controls. (**a**) Decreased GAS5 level in RA patients compared with controls; (**b**) Decreased linc0597 level in RA patients compared with controls; (**c**) Decreased H19 level in RA patients compared with controls. Data for lncRNAs expression levels were presented as median (interquartile range) (* *p* < 0.05, *** *p* < 0.001). RA, rheumatoid arthritis; HC, controls; PBMCs, peripheral blood mononuclear cells.

The expression levels of selected lncRNAs (H19, GAS5 and linc0597) were significantly decreased in PBMCs from RA patients compared with controls (GAS5, *Z* = −4.821, *p* < 0.001; linc0597, *Z* = −6.095, *p* < 0.001; H19, *Z* = −2.330, *p* = 0.020) (Figure 1).

The correlations between H19, GAS5 and linc0597 levels and major laboratory parameters or disease activity of RA patients were further analyzed (Table 2 and Table 3). GAS5 level was down-regulated in RA patients with hypocomplementemia than those with normal levels of complements (*Z* = −2.259, *p* = 0.024). Correlation analysis demonstrated that GAS5 level was negatively associated with C-reactive protein (CRP) level (*r_s_*= −0.273, *p* = 0.017). We found no associations of H19 and linc0597 levels with laboratory parameters (all *p* > 0.05). Likewise, these three lncRNAs were not correlated with disease activity of RA patients (all *p* > 0.05).

When the potential effects of medical therapies on lncRNAs expression were considered, we observed that none of RA patients treated for immunosuppressive therapy. Expression of H19, GAS5 and linc0597 exhibited no statistical differences between RA patients who treated with medium to high doses of prednisone (>7.5 mg/day) and those who treated with low doses of prednisone (≤7.5 mg/day) (all *p* > 0.05). Compared with RA patients treated with any of the following disease modifying antirheumatic drugs (DMARDS): hydroxychloroquine, methotrexate, leflunomide or sulfasalazine, expression of the three lncRNAs showed no differences in RA patients who have not received treatment with DMARDS either (all *p* > 0.05). Similarly, the three lncRNAs showed no differences between RA patients who have received botanical preparation treatment or not, such as total glucosides of paeony (all *p* > 0.05) (Table 4).

### 3.2. Association between lncRNAs Gene Single Nucleotide Polymorphisms and RA Susceptibility

Fourteen SNPs (rs2067051 and rs2075745 for H19; rs6790, rs16847206, and rs6692753 for GAS5; rs2070107, rs2877877, rs2632516, rs2285991, rs8071916, rs12601867, rs13414, rs2680700, and rs4372750 for linc0597) were selected to detect the correlation of lncRNAs related gene polymorphisms with RA susceptibility in 828 RA patients and 780 controls. Our results demonstrated that the genotype frequencies of rs6790 and rs2680700 were associated with RA risk (all *p* < 0.05) (Table 5). However, we failed to observe the associations of lncRNAs gene variation with RA risk under dominant models and recessive models (all *p* > 0.05).

We further analyzed the correlations of lncRNAs SNPs with major clinical features in RA patients (Table S3). There were associations between rs2285991 and anti-cyclic citrullinated peptide (anti -CCP) and rheumatoid factor (RF) (all *p* < 0.05).

### 3.3. Association of lncRNAs Expression Levels with Their Gene Single Nucleotide Polymorphisms in RA Patients

The associations between H19, GAS5 and linc0597 expression and genotypes in RA patients were analyzed, and the results showed that rs4372750 was correlated with GAS5 expression level (Table 6).

## 4. Discussion

RA is a complex autoimmune disease resulting from multiple factors, as well as a clinical syndrome spanning several disease subsets [1]. To the best of our knowledge, almost all of RA disease subsets present persistent synovial inflammation, associated bone, and articular cartilage damage [1]. These disorders complicated with extra-articular diseases can damage any part of the body [3]. Recent research has advanced the understanding of RA pathogenesis to the point where lncRNAs biological functions for RA pathogenetic process are underway, and the excavation of novel functions of thousands of lncRNAs promoted us to explore their affects in RA.

In the present study, we investigated three lncRNAs (H19, GAS5 and linc0597) expression profiles in PBMCs of patients with RA by qT-PCR in the first stage, and further analyzed the correlations between their expression and laboratory parameters of RA patients. The results demonstrated that the expression levels of H19, GAS5 and linc0597 were lower in RA patients. H19 could exert carcinogenesis as precursor of miRNA or ceRNA, such as precursor for miR-675 and modulating let-7 family of miRNAs [29]. Metabolic stress-induced H19 and its encoded miR-675 could regulate inflammatory and hypoxic conditions by directly affecting COL2A1 [30]. The expression of H19 in RA synovial fibroblasts was not only induced by cytokines/serum starvation but also correlated with mRNA expression for TIMP-2 [22]. Taken together, it is fully suggested that H19 may play a pathogenic role in RA. However, Stuhlmüller et al. reported that H19 was overexpressed in RA synovial tissues [22]. Song et al. indicated that H19 antisense expression was higher in PBMCs of patients with RA [31]. However, our study showed that the expression level of H19 decreased in PBMCs of patients with RA. The possible explanation is that different locations in the genome of this lncRNA or lncRNAs expression possess tissue specificity. In addition, we also studied GAS5 in patients with RA. Indeed, related research on several other autoimmune diseases has shown its vital roles in a wide range of biological processes. GAS5 was reported to bind glucocorticoid receptor (GR) DNA-binding domain (DBD) and suppress GR-induced transcriptional activity, thereby inducing apoptosis [32]. Most importantly, GAS5 was demonstrated to promote apoptosis and trigger growth arrest in human T-cells [33]. All of the above are implicated in the pathogenesis of RA [34]. Moreover, negative correlations between GAS5 expression and CRP level and hypocomplementemia in patients with RA were also found in our study. CRP is a product of inflammatory response, playing pro-inflammatory roles in RA by activating complements and inducing osteoclast differentiation, and acts as an indicator of treatment efficacy [35]. Besides, complement system plays central roles in numerous protective immune processes [36]. Complement C3 and C4 are also one of the most important indicators of RA disease activity [36]. Thus, we speculate that GAS5 may play a pivotal role in the pathogenesis of RA, and its underlying mechanism is worth further exploration. Furthermore, it is generally acknowledged that TNF-α and IL-6 are documented in RA pathogenesis [37,38]. Linc0597 expression level was lower in THP1 macrophages and might regulate the induction of pro-inflammatory TNF-α and IL-6 [39]. However, linc0597, which was previously found to be highly expressed in PBMCs of patients with RA [23], showed low expression level in our study. One explanation is that a different internal control was used between two studies. The other explanation is that linc0597 expression may be affected by clinical treatment.

Then, gene polymorphisms of differentially expressed lncRNAs (rs2067051 and rs2075745 for H19; rs6790, rs16847206, and rs6692753 for GAS5; rs2070107, rs2877877, rs2632516, rs2285991, rs8071916, rs12601867, rs13414, rs2680700, and rs4372750 for linc0597) with RA susceptibility were detected in the second stage. Among these fourteen SNPs, rs12601867, rs13414, rs2680700, rs4372750, rs16847206, rs8071916, and rs6692753 were first reported in this study, and rs6790 genotype (GA) and rs2680700 genotype (GT) were found to be associated with RA risk. Previous studies prompted that certain functional SNPs within the promoter region of GAS5 could regulation its expression, and were related to risk of cancers, among which rs6790 were suggested as a biomarker for chemoradiotherapy induced toxic reactions in nasopharyngeal cancer patients [40,41,42]. CHIP-seq data showed that rs6790 was labeled as an active promoter region or enhancer region position, and silico analysis suggested that this SNP also possessed a significant feature of e-QTL, both of them verified its contribution in the genetic variance of GAS5 expression [41]. Similarly, rs6790 was reported to play inhibitory functions of GAS5 on the transcriptional activity of GR in autoimmune disease [43], GAS5 knock-down could attenuate the progression of experimental autoimmune encephalomyelitis (EAE) and promote remyelination [44]. Since no studies reported on linc0597 rs2680700, we tried to explore its potential functions through bioinformatics databases. rs2680700 is also labeled as an enhancer region according to ChIP-seq data, and it has a strong feature of e-QTL in several tissues (Table S4), suggesting that rs2680700 might exert their roles through affecting the expression of target genes. Taken together, these data provide evidence for functional contribution of rs2680700 in changing linc0597 expression or stability. However, the exact roles of these two SNPs in RA still need further exploration [45,46]. Additionally, we attempted to discover the correlations of H19 expression with their gene polymorphisms in RA patients, but still no statistically significant results were found. Similarly, Huang et al. and Zhou et al. showed that H19 (rs2839698, rs3741219 and rs217727) gene polymorphisms were not related to RA risk in a Chinese population [47,48], indicating that there might be other mechanisms for H19 in RA pathogenesis.

Presently, more and more studies reveal the impact of lncRNAs on the pathogenesis of RA. There are evidence that lncRNAs regulate sphingomyelin phosphodiesterase 1 (SMPD1), and SMPD1 can regulate differentiation and apoptosis in T-cells [49]. Additionally, Hotair contributes to RA pathogenesis through activation of matrix metalloproteinases 2 (MMP-2) and MMP-13 in synoviocytes and osteoclasts [31], and MMPs play vital roles in recruitment of inflammatory cells and degradation of cartilage and bone of RA [50]. Linc-p21 was confirmed to be negative regulators of NF-κB activity, and NF-κB is a pro-inflammatory transcription factor of RA patients [51]. At present, emerging evidence proved that lncRNAs might regulate transcript levels of genes in the same genomic region, for instance, lncRNA C5T1 located in TRAF1-C5 influenced C5 mRNA level in RA risk [52]. Last but not least, the regulation of lncRNAs might be specific in cells of innate immune system [53]. For instance, Müller et al. reported that 7419 lncRNAs were detected in CD14+ monocytes isolated before and after IL-6 or TNF-α inhibition in RA, among them, only 85 lncRNAs examined exhibited up-regulation and down-regulation by anti-cytokine treatment initiated, and none of lncRNAs identified showed a similar pattern in response to IL-6 versus TNF-α inhibition [53]. Overall, lncRNAs are emerging as key regulators of activation, differentiation, and expression of immune cells, which may direct or indirect link to cellular and tissue homeostasis of autoimmunity [54,55].

Several limitations should be acknowledged in our study. Due to the lack of data on non-steroidal anti-inflammatory drug (NSAIDs), the effects of NSAIDs on the expression of lncRNAs in RA patients might not be known. In addition, this is a hospital-based case-control study, so that a causal relationship was difficultly determined between RA risk and lncRNAs expression.

## 5. Conclusions

Our study discovered down-regulated expression of H19, linc0597 and GAS5 in RA patients, GAS5 level is correlated with complement and CRP of RA patients. Moreover, we highlighted two related potential functional locus, GAS5 rs6790 and linc0597 rs2680700 for associations with RA susceptibility. Future studies are still needed to further explore the exact roles of these lncRNAs in the development and pathogenesis of RA.

## Figures and Tables

**Table 1:** Primers used in real time quantitative PCR experiment.

RNAs	Primers
H19	F:5′TGCTGCACTTTACAACCACTG3′
	R:5′ATGGTGTCTTTGATGTTGGGC3′
GAS5	F:5′TATGGTGCTGGGTGCGGAT3′
	R:5′CCAATGGCTTGAGTTAGGCTT3′
linc0597	F:5′TTGGATTCATCCCGTTCACCTCCA3′
	R:5′CAGCATGACGATCAAGCGAGATTC3′
β-actin	F:5′CACGAAACTACCTTCAACTCC3′
	R:5′CATACTCCTGCTTGCTGATC3′

**Table 2:** Associations between lncRNAs expression and categorical laboratory parameters in RA patients.

Parameters	Number	GAS5	linc0597	H19
RF				
Positive	69	0.50 (0.34, 0.72)	0.62 (0.47, 0.78)	0.56 (0.35, 0.82)
Negative	8	0.52 (0.33, 0.92)	0.54 (0.34, 0.86)	0.57 (0.16, 1.18)
Anti-CCP				
Positive	65	0.51 (0.35, 0.74)	0.61 (0.47, 0.79)	0.61 (0.35, 0.83)
Negative	12	0.34 (0.25, 0.77)	0.63 (0.37, 0.73)	0.36 (0.17, 0.74)
Low complement				
Positive	14	0.35 (0.21, 0.46)	0.50 (0.41, 0.69)	0.52 (0.27, 1.23)
Negative	52	0.53 (0.34, 0.77)	0.62 (0.53, 0.80)	0.60 (0.36, 0.82)

Anti-CCP, anti-cyclic citrullinated peptide; RF, rheumatoid factor; RA, rheumatoid arthritis.

**Table 3:** Correlations of lncRNAs expression with continuous laboratory parameters and degree of disease activity in RA patients.

Parameters	GAS5		linc0597		H19	
	*r_s_*	*p* Value	*r_s_*	*p* Value	*r_s_*	*p* Value
ESR	−0.072	0.535	−0.114	0.325	−0.150	0.196
CRP	−0.273	0.017	−0.096	0.411	0.002	0.986
TJC	−0.020	0.866	−0.091	0.435	−0.192	0.097
SJC	−0.054	0.648	0.052	0.660	−0.110	0.924
DAS28-ESR	−0.112	0.337	−0.012	0.920	−0.166	0.153

DAS28-ESR, 28-joint disease activity score using erythrocyte sedimentation rate; CRP, C-reaction. protein; ESR, erythrocyte sedimentation rate; TJC, tender joint count; SJC, swollen joint count.

**Table 4:** Influence of main clinical medication on lncRNAs expression in RA patients.

Group	Number	GAS5	linc0597	H19
Prednisone (mg/day)				
≤7.5	36	0.61 (0.37, 0.94)	0.61 (0.46, 0.81)	0.58 (0.30, 0.87)
>7.5	37	0.42 (0.34, 0.68)	0.62 (0.48, 0.78)	0.55 (0.31, 0.82)
DMARDS				
Yes	44	0.51 (0.36, 0.81)	0.62 (0.47, 0.78)	0.61 (0.34, 0.84)
No	29	0.53 (0.34, 0.69)	0.59 (0.47, 0.80)	0.43 (0.29, 0.84)
Botanical preparation				
Yes	22	0.51 (0.36, 0.84)	0.63 (0.46, 0.82)	0.59 (0.46, 1.13)
No	51	0.53 (0.34, 0.76)	0.62 (0.48, 0.79)	0.49 (0.27, 0.80)

DMARDS, disease modifying antirheumatic drugs.

**Table 5:** Allele and genotype frequencies of fourteen SNPs in RA patients and controls.

SNPs	Analysis Model	RA *n*(%)	HC *n*(%)	*χ* ^2^	*OR* (95% *CI*)	*p*_ad__just_ Value
rs6790	Genotype					
	GG	362 (43.9)	316 (40.8)	1.103	0.848 (0.623–1.154)	0.294
	GA	333 (40.4)	365 (47.1)	6.132	0.679 (0.499–0.922)	0.013
	AA	130 (15.8)	94 (12.1)		1.000	
	Allele					
	G	1057 (63.9)	997 (63.4)	0.024	0.989 (0.856–1.142)	0.877
	A	593 (36.1)	553 (36.6)		1.000	
	Dominant model					
	AA + GA	463 (56.1)	459 (59.2)	1.630	0.878 (0.719–1.072)	0.202
	GG	362 (43.9)	316 (40.8)		1.000	
	Recessive model					
	AA	130 (15.8)	94 (12.1)	3.578	1.321 (0.990–1.762)	0.059
	GA + GG	695 (84.2)	681 (87.9)		1.000	
rs2067051	Genotypes					
	TT	101 (12.4)	80 (10.7)	1.059	1.189 (0.855–1.653)	0.303
	TC	335 (41.1)	306 (41.0)	0.140	1.042 (0.842–1.289)	0.708
	CC	380 (46.6)	360 (48.3)		1.000	
	Allele					
	T	537 (33.0)	466 (31.6)	0.998	1.080 (0.929–1.255)	0.318
	C	1095 (67.0)	1026 (68.4)		1.000	
	Dominant model					
	TT + TC	436 (53.4)	386 (51.7)	0.464	1.072 (0.878–1.130)	0.496
	CC	380 (46.6)	360 (48.3)		1.000	
	Recessive model					
	TT	101 (12.4)	80 (10.7)	0.923	1.167 (0.852–1.598)	0.337
	TC + CC	715 (87.6)	666 (89.3)		1.000	
rs2070107	Genotype					
	GG	576 (69.2)	549 (72.0)	0.022	0.955 (0.522–1.747)	0.881
	GC	233 (28.0)	193 (25.3)	0.089	1.100 (0.590–2.050)	0.765
	CC	23 (2.8)	21 (2.8)			
	Allele					
	G	1385(83.2)	1291(84.6)	1.100	0.904 (0.748–1.092)	0.294
	C	279 (16.8)	235 (15.4)		1.000	
	Dominant model					
	GC + CC	256 (30.8)	214 (28.0)	1.424	1.141 (0.919–1.418)	0.233
	GG	576 (69.2)	549 (72.0)		1.000	
	Recessive model					
	CC	23 (2.8)	21 (2.8)	0.001	1.008(0.553–1.838)	0.980
	GC + GG	809 (97.2)	742 (97.2)			
rs2075745	Genotype					
	TT	113 (14.1)	106 (14.2)	0.080	0.956 (0.703–1.302)	0.777
	TA	340 (42.3)	318 (42.6)	0.026	0.982 (0.791–1.219)	0.871
	AA	350 (43.6)	323 (43.2)		1.000	
	Allele					
	T	566 (35.2)	530 (35.5)	0.018	0.990 (0.854–1.147)	0.892
	A	1040 (64.8)	964 (64.5)		1.000	
	Dominant model					
	TT + TA	453 (56.4)	424 (56.8)	0.056	0.976 (0.797–1.194)	0.812
	AA	350 (43.6)	323 (43.2)		1.000	
	Recessive model					
	TT	113 (14.1)	106 (14.2)	0.058	0.965(0.722–1.289)	0.809
	TA + AA	690 (85.9)	641 (85.8)		1.000	
rs2285991	Genotype					
	GG	678 (86.1)	612 (86.6)	0.773	0.611 (0.203–1.834)	0.379
	GA	100 (12.7)	90 (12.7)	0.719	0.613 (0.198–1.900)	0.396
	AA	9 (1.1)	5 (0.7)		1.000	
	Allele					
	G	1456 (92.5)	1314 (92.9)	0.199	0.939 (0.712–1.238)	0.656
	A	118 (7.5)	100 (7.1)		1.000	
	Dominant model					
	GA + AA	109 (13.9)	95 (13.4)	0.058	1.037 (0.770–1.397)	0.809
	GG	678 (86.1)	612 (86.6)		1.000	
	Recessive model					
	AA	9 (1.1)	5 (0.7)	0.772	1.637 (0.545–4.914)	0.380
	GA + GG	778 (98.9)	702 (99.3)		1.000	
rs2632516	Genotype					
	GG	270 (32.6)	229 (29.4)	1.151	1.161 (0.884–1.527)	0.283
	GC	379 (45.8))	373 (47.8)	0.015	1.016 (0.789–1.309)	0.901
	CC	179 (21.6)	178 (22.8)		1.000	
	Allele					
	G	919 (55.5)	831 (53.3)	1.604	1.094 (0.952–1.257)	0.205
	C	737 (44.5)	729 (46.7)		1.000	
	Dominant model					
	GG + GC	649 (78.4)	602 (77.2)	0.329	1.072 (0.846–1.357)	0.566
	CC	179 (21.6)	178 (22.8)		1.000	
	Recessive model					
	GG	270 (32.6)	229 (29.4)	1.632	1.149 (0.928–1.422)	0.201
	GC + CC	558 (67.4)	551 (70.6)		1.000	
rs2877877	Genotype					
	GG	50 (6.1)	53 (7.3)	1.270	0.790 (0.524–1.190)	0.260
	GA	272 (33.1)	248 (34.3)	0.561	0.921 (0.741–1.143)	0.454
	AA	500 (60.8)	422 (58.4)		1.000	
	Allele					
	G	372 (22.6)	354 (24.5)	1.470	0.902 (0.764–1.066)	0.225
	A	1272 (77.4)	1092 (75.5)		1.000	
	Dominant model					
	GG + GA	322 (39.2)	301 (41.6)	1.070	0.898 (0.731–1.101)	0.301
	AA	500 (60.8)	422 (58.4)		1.000	
	Recessive model					
	GG	50 (6.1)	53 (7.3)	1.006	0.814 (0.544–1.217)	0.316
	GA + AA	772 (93.9)	670 (92.7)		1.000	
rs13414	Genotype					
	AA	368 (51.0)	316 (46.7)	0.149	1.076 (0.741–1.563)	0.700
	AG	283 (39.3)	295 (43.6)	0.403	0.885 (0.606–1.292)	0.526
	GG	70 (9.7)	65 (9.6)		1.000	
	Allele					
	A	1019 (70.7)	927 (68.6)	1.456	1.104 (0.940–1.298)	0.228
	G	423 (29.3)	425 (31.4)		1.000	
	Dominant model					
	AG + GG	353 (49.0)	360 (53.3)	2.551	0.841 (0.681–1.040)	0.110
	AA	368 (51.0)	316 (46.7)		1.000	
	Recessive model					
	GG	70 (9.7)	65 (9.6)	0.008	1.016 (0.710–1.455)	0.930
	AG + AA	651 (90.3)	611 (90.4)		1.000	
rs4372750	Genotype					
	AA	168 (23.5)	156 (23.0)	0.184	1.070 (0.786–1.456)	0.668
	AC	378 (52.8)	352 (51.9)	0.370	1.084 (0.836–1.405)	0.543
	CC	170 (23.7)	170 (25.1)		1.000	
	Allele					
	A	714 (49.9)	664 (49.0)	0.222	1.036 (0.893–1.202)	0.637
	C	718 (50.1)	692 (51.0)		1.000	
	Dominant model					
	AA + AC	546 (76.3)	508 (74.9)	0.318	1.074 (0.839–1.374)	0.573
	CC	170 (23.7)	170 (25.1)		1.000	
	Recessive model					
	AA	168 (23.5)	156 (23.0)	0.006	1.007 (0.999–1.015)	0.937
	AC+CC	548 (76.5)	522 (77.0)		1.000	
rs12601867	Genotype					
	CC	165 (22.6)	166 (24.0)	0.953	0.860 (0.634–1.165)	0.329
	CG	381 (52.1)	361 (52.2)	0.274	0.934 (0.723–1.206)	0.601
	GG	185 (25.3)	165 (23.8)		1.000	
	Allele					
	C	711 (48.6)	693 (50.1)	0.590	0.944 (0.815–1.094)	0.442
	G	751 (51.4)	691 (49.9)		1.000	
	Dominant model					
	CG + GG	566 (77.4)	526 (76.0)	0.681	1.111 (0.866–1.425)	0.409
	CC	165 (22.6)	166 (24.0)		1.000	
	Recessive model					
	GG	185 (25.3)	165 (23.8)	0.571	1.098 (0.861–1.400)	0.450
	CG + CC	546 (74.7)	527 (76.2)		1.000	
rs16847206	Genotype					
	AA	338 (46.5)	296 (43.7)	1.358	1.247 (0.860–1.808)	0.244
	AT	325 (44.7)	308 (45.5)	0.679	1.169 (0.806–1.694)	0.410
	TT	64 (8.8)	73 (10.8)		1.000	
	Allele					
	A	1001 (68.8)	900 (66.5)	1.808	1.115 (0.952–1.306)	0.179
	T	453 (31.2)	454 (33.5)		1.000	
	Dominant model					
	AT + TT	389 (53.5)	381 (56.3)	0.735	0.911 (0.737–1.127)	0.391
	AA	338 (46.5)	296 (43.7)		1.000	
	Recessive model					
	TT	64 (8.8)	73 (10.8)	1.086	0.828 (0.581–1.180)	0.297
	AT + AA	663 (91.2)	604 (89.2)			
rs6692753	Genotype					
	GG	339 (46.6)	287 (42.0)	1.624	1.272 (0.878–1.843)	0.203
	GT	323 (44.4)	323 (47.3)	0.241	1.097 (0.758–1.587)	0.623
	TT	65 (8.9)	73 (10.7)		1.000	
	Allele					
	G	1001 (68.8)	897 (65.7)	3.231	1.155 (0.987–1.352)	0.072
	T	453 (31.2)	469 (34.3)		1.000	
	Dominant model					
	GT + TT	388 (53.4)	396 (58.0)	2.315	0.848 (0.686–1.049)	0.128
	GG	339 (46.6)	287 (42.0)		1.000	
	Recessive model					
	TT	65 (8.9)	73 (10.7)	0.842	0.848 (0.596–1.206)	0.359
	GT + GG	662 (91.1)	610 (89.3)		1.000	
rs2680700	Genotype					
	GG	412 (57.3)	376 (55.1)	1.773	0.755 (0.499–1.142)	0.183
	GT	243 (33.8)	263 (38.6)	4.477	0.630 (0.411–0.967)	0.034
	TT	64 (8.9)	43 (6.3)		1.000	
	Allele					
	G	1067 (74.2)	1015 (74.4)	0.017	0.989 (0.835–1.172)	0.897
	T	371 (25.8)	349 (25.6)		1.000	
	Dominant model					
	GT + TT	307 (42.7)	306 (44.9)	0.857	0.904 (0.731–1.119)	0.355
	GG	412 (57.3)	376 (55.1)		1.000	
	Recessive model					
	TT	64 (8.9)	43 (6.3)	2.896	1.421 (0.948–2.131)	0.089
	GT+GG	655 (91.1)	639 (93.7)		1.000	
rs8071916	Genotype					
	AA	175 (24.1)	162 (23.4)	0.016	1.020 (0.751–1.384)	0.900
	AG	378 (52.0)	366 (53.0)	0.015	0.984 (0.758–1.277)	0.904
	GG	174 (23.9)	163 (23.6)		1.000	
	Allele					
	A	728 (50.1)	690 (49.9)	0.006	1.006 (0.868–1.165)	0.940
	G	726 (49.9)	692 (50.1)		1.000	
	Dominant model					
	AG + GG	552 (75.9)	529 (76.6)	0.059	0.970 (0.758–1.241)	0.808
	AA	175 (24.1)	162 (23.4)		1.000	
	Recessive model					
	GG	174 (23.9)	163 (23.6)	0.002	1.005 (0.785–1.287)	0.969
	AG + AA	553 (76.1)	528 (76.4)		1.000	

RA, rheumatoid arthritis; HC, control; OR, odds ratio; CI, confidence interval; SNPs, single nucleotide polymorphisms; *p*_adjust_, *p* value adjusted for gender and age.

**Table 6:** Correlations of lncRNAs expression levels with genotypes of gene single nucleotide polymorphisms in RA patients.

SNPs	GAS5		linc0597		H19	
	*r_s_*	*p* Value	*r_s_*	*p* Value	*r_s_*	*p* Value
rs2067051	−0.030	0.823	−0.076	0.569	−0.011	0.935
rs2075745	−0.025	0.850	−0.055	0.681	−0.107	0.420
rs2877877	0.085	0.523	0.088	0.509	−0.031	0.815
rs2070107	−0.077	0.560	−0.142	0.285	−0.051	0.703
rs2632516	0.096	0.470	−0.094	0.478	0.031	0.816
rs6790	−0.010	0.941	0.029	0.828	0.068	0.611
rs2285991	0.103	0.438	−0.079	0.551	0.055	0.677
rs13414	−0.149	0.360	−0.041	0.803	0.014	0.930
rs4372750	0.344	0.030	−0.010	0.950	0.202	0.211
rs12601867	0.216	0.180	0.010	0.950	0.244	0.129
rs16847206	−0.135	0.414	−0.090	0.588	−0.136	0.408
rs6692753	−0.143	0.378	−0.100	0.538	−0.145	0.373
rs2680700	−0.149	0.358	−0.135	0.406	−0.013	0.939
rs8071916	−0.279	0.081	−0.027	0.870	−0.250	0.120

SNPs, gene single nucleotide polymorphisms.

## Supplementary Materials

The following are available online at https://www.mdpi.com/2218-273X/10/1/55/s1, Table S1: Characteristics of RA patients and controls in the stage of lncRNAs expression detection, Table S2: Characteristics of RA patients and controls in the stage of genotyping, Table S3: Associations of fourteen SNPs with risk of different serotypes in RA patients, Table S4: e-QTLs effects of rs2680700 and rs6790 in multi-tissues.
