# Supplementary material for: Decreased H19, GAS5, and linc0597 Expression and Association Analysis of Related Gene Polymorphisms in Rheumatoid Arthritis
Source: Biomolecules. 2019 Dec 29;10(1):55. doi: 10.3390/biom10010055 (PMC7022387; doi:10.3390/biom10010055)
Supplement: Supplementary file 1 [file biomolecules-10-00055-s001.pdf]

Table S1. Characteristics of RA patients and controls in the stage of lncRNAs expression detection

Characteristics	RA(n=77)	HC(n=78)	χ^2 / t	P
Sex n(%)			0.002	0.965
Male	18（23.4）	18（23.1）		
Female	59（76.6）	60（76.9）		
Age (y) (mean ± SD)	58.0±13.2	56.0±10.6	1.047	0.297

y, years; RA, rheumatoid arthritis; HC, control, SD, standard deviation.

Table S2. Characteristics of RA patients and controls in the stage of genotyping

Characteristics	SNPs	Number		RA	HC	t/χ^2	P value	P_{HWE}(HC) value
		RA	HC					
Sex(male/female)	rs2067051	816	746	125/691	141/605	3.539	>0.05	>0.05
Age (y)				53.2±13.1	52.5±13.9	-0.890	>0.05	
Sex(male/female)	rs2075745	803	747	122/681	133/614	1.920	>0.05	>0.05
Age (y)				53.1±13.1	52.5±14.1	-0.780	>0.05	
Sex(male/female)	rs2877877	822	723	128/694	130/593	1.605	>0.05	>0.05
Age (y)				53.2±13.0	52.3±14.0	-1.281	>0.05	
Sex(male/female)	rs2070107	832	763	126/706	143/620	3.674	>0.05	>0.05
Age (y)				53.0±13.0	52.6±14.0	-0.485	>0.05	
Sex(male/female)	rs2632516	828	780	128/700	149/631	3.740	>0.05	>0.05
Age (y)				53.0±13.0	52.7±14.0	-0.434	>0.05	
Sex(male/female)	rs6790	825	775	127/698	146/629	3.351	>0.05	>0.05
Age (y)				53.2±13.1	52.9±13.9	-0.370	>0.05	
Sex(male/female)	rs2285991	787	707	118/669	132/575	3.613	>0.05	>0.05
Age (y)				52.9±13.0	52.8±13.9	-0.088	>0.05	
Sex(male/female)	rs13414	720	676	110/610	129/547	3.558	>0.05	>0.05
Age (y)				53.4±12.9	52.5±14.1	-1.178	>0.05	
Sex(male/female)	rs4372750	715	678	109/606	129/549	3.513	>0.05	>0.05
Age (y)				53.3±12.9	52.4±14.2	-1.226	>0.05	
Sex(male/female)	rs12601867	731	692	112/619	132/560	3.526	>0.05	>0.05
Age (y)				53.4±12.9	52.4±14.3	-1.329	>0.05	
Sex(male/female)	rs16847206	727	677	110/617	129/548	3.776	>0.05	>0.05
Age (y)				53.3±12.9	52.5±14.1	-1.033	>0.05	
Sex(male/female)	rs6692753	726	683	110/616	129/554	3.487	>0.05	>0.05
Age (y)				53.2±12.9	52.4±14.3	-1.120	>0.05	
Sex(male/female)	rs2680700	720	683	111/609	132/551	3.755	>0.05	>0.05
Age (y)				53.2±12.9	52.4±14.2	-1.078	>0.05	
Sex(male/female)	rs8071916	726	692	109/617	131/561	3.645	>0.05	>0.05
Age (y)				53.3±12.9	51.4±14.2	-1.210	>0.05	

Abbreviation: y, years; RA, rheumatoid arthritis; HC, control; P_{HWE}, P-value for Hardy-Weinberg equilibrium.

Table S3. Associations of fourteen SNPs with risk of different serotypes in RA patients

SNPs	Allele (M/m)	Clinical features	Group	Genotype n (%)			P_{adjust} value	Allele n (%)		P value
				MM	Mm	mm		M	m	
rs6790	G/A	Anti-CCP	Positive	298(43.4)	280(40.8)	109(15.9)	0.701	876(63.8)	498(36.2)	0.419
			Negative	47(46.1)	42(41.2)	13(12.7)		136(66.7)	68(33.3)	
		RF	Positive	298(43.8)	275(40.4)	107(15.7)	0.963	871(64.0)	489(36.0)	0.831
			Negative	54(44.3)	50(41.0)	18(14.8)		158(64.8)	86(35.2)	
rs2067051	C/T	Anti-CCP	Positive	322(47.4)	276(40.6)	82(12.1)	0.279	920(67.6)	440(32.4)	0.545
			Negative	41(41.0)	49(49.0)	10(10.0)		131(65.5)	69(34.5)	
		RF	Positive	309(46.0)	275(40.9)	88(13.1)	0.471	893(66.4)	451(33.6)	0.365
			Negative	58(47.9)	52(43.0)	11(9.1)		168(69.4)	74(30.6)	
rs2075745	A/T	Anti-CCP	Positive	299(44.8)	274(41.1)	94(14.1)	0.118	872(65.4)	462(34.6)	0.352
			Negative	36(36.0)	52(52.0)	12(12.0)		124(62.0)	76(38.0)	
		RF	Positive	286(43.3)	277(41.9)	98(14.8)	0.527	849(64.2)	473(35.8)	0.442
			Negative	53(44.5)	53(44.5)	13(10.9)		159(66.8)	79(33.2)	
rs2877877	A/G	Anti-CCP	Positive	411(60.1)	230(33.6)	43(6.3)	0.851	1052(76.9)	316(23.1)	0.627
			Negative	63(61.8)	34(33.3)	5(4.9)		160(78.4)	44(21.6)	
		RF	Positive	414(60.9)	224(32.9)	42(6.2)	0.744	1052(77.4)	308(22.6)	0.468
			Negative	68(57.1)	43(36.1)	8(6.7)		179(75.2)	59(24.8)	
rs2070107	G/C	Anti-CCP	Positive	483(69.7)	190(27.4)	20(2.9)	0.739	1156(83.4)	230(16.6)	0.507
			Negative	68(66.0)	32(31.1)	3(2.9)		168(81.6)	38(18.4)	
		RF	Positive	482(70.3)	184(26.8)	20(2.9)	0.180	1148(83.7)	224(16.3)	0.166
			Negative	77(62.6)	43(35.0)	3(2.4)		197(80.1)	49(19.9)	
rs2632516	C/G	Anti -CCP	Positive	154(22.4)	307(44.6)	228(33.1)	0.423	615(44.6)	763(55.4)	0.890
			Negative	20(19.4)	53(51.5)	30(29.1)		93(45.1)	113(54.9)	
		RF	Positive	149(21.8)	303(44.4)	231(33.8)	0.118	601(44.0)	765(56.0)	0.134
			Negative	28(23.0)	64(52.5)	30(24.6)		120(49.2)	124(50.8)	

rs2285991	G/A	Anti -CCP	Positive	577(87.4)	75(11.4)	8(1.2)	0.107	1299(93.1)	91(6.9)	<0.001
			Negative	77(81.9)	17(18.1)	0(0.0)		171(91.0)	31(9.0)	
		RF	Positive	560(86.3)	80(12.3)	9(1.4)	0.411	1200(92.4)	98(7.6)	0.002
			Negative	100(86.2)	16(13.8)	0(0.0)		216(86.4)	34(13.6)	
rs13414	A/G	Anti-CCP	Positive	296(50.5)	235(40.1)	55(9.4)	0.777	827(70.6)	345(29.4)	0.760
			Negative	48(50.5)	36(37.9)	11(11.6)		132(69.5)	58(30.5)	
		RF	Positive	305(50.7)	239(39.7)	58(9.6)	0.912	849(70.5)	355(29.5)	0.971
			Negative	53(51.5)	39(37.9)	11(10.7)		145(70.4)	61(29.6)	
rs4372750	C/A	Anti-CCP	Positive	138(23.8)	304(52.4)	138(23.8)	0.839	580(50.0)	580(50.0)	0.687
			Negative	20(21.1)	52(54.7)	23(24.2)		92(48.4)	98(51. 6)	
		RF	Positive	146(24.5)	310(52.1)	139(23.4)	0.470	602(50.6)	588(49.4)	0.295
			Negative	20(19.0)	58(55.2)	27(25.7)		98(46.7)	112(53.3)	
rs12601867	C/G	Anti-CCP	Positive	135(22.7)	311(52.4)	148(24.9)	0.872	581(48.9)	607(51.1)	0.991
			Negative	23(24.2)	47(49.5)	25(26.3)		93(48.9)	97(51.1)	
		RF	Positive	136(22.4)	315(51.8)	157(25.8)	0.752	587(48.3)	629(51.7)	0.473
			Negative	27(25.5)	54(50.9)	25(23.6)		108(50.9)	104(49.1)	
rs16847206	A/T	Anti-CCP	Positive	268(45.4)	269(45.6)	53(9.0)	0.629	805(68.2)	375(31.8)	0.354
			Negative	48(50.5)	40(42.1)	7(7.4)		136(71.6)	54(28.4)	
		RF	Positive	280(46.2)	273(45.0)	53(8.7)	0.864	833(68.7)	379(31.3)	0.885
			Negative	50(48.1)	44(42.3)	10(9.6)		144(69.2)	64(30.8)	
rs6692753	G/T	Anti-CCP	Positive	269(45.5)	267(45.2)	55(9.3)	0.439	805(68.1)	377(31.9)	0.212
			Negative	49(51.6)	40(42.1)	6(6.3)		138(72.6)	52(27.4)	
		RF	Positive	278(46.0)	273(45.2)	53(8.8)	0.512	829(68.6)	379(31.4)	0.669
			Negative	54(50.5)	42(39.3)	11(10.3)		150(70.1)	64(29.9)	
rs2680700	G/T	Anti-CCP	Positive	328(56.0)	206(35.2)	52(8.9)	0.401	862(73.5)	310(26.5)	0.202
			Negative	59(63.4)	27(29.0)	7(7.5)		145(80.0)	41(22.0)	
		RF	Positive	346(57.8)	198(33.1)	55(9.2)	0.439	890(74.3)	308(25.7)	0.607
			Negative	55(52.9)	41(39.4)	8(7.7)		151(72.6)	57(27.4)	
rs8071916	A/G	Anti-CCP	Positive	140(23.7)	305(51.7)	145(24.6)	0.991	585(49.6)	595(50.4)	0.913
			Negative	23(24.0)	50(52.1)	23(24.0)		96(50.0)	96(50.0)	
		RF	Positive	147(24.3)	312(51.7)	145(24.0)	0.904	606(50.2)	602(49.8)	0.673
			Negative	24(22.4)	56(52.3)	27(25.2)		104(48.6)	110(51.4)	

Anti-CCP, anti-cyclic citrullinated peptide; RF, rheumatoid factor; M, major alleles; m, minor alleles; a, Fisher's exact test; SNP, single nucleotide polymorphism; P_{adjust}, P-value adjusted for gender and age.

Table S4. e-QTLs effects of rs2680700 and rs6790 in multi-tissues.

Gencode Id	Gene	*P* value	Effect size	Tissue
eQTLs of rs2680700 in multi-tissue				
ENSG00000108395.13	TRIM37	3.30E-15	0.29	Whole Blood
ENSG00000108384.14	RAD51C	4.80E-09	0.22	Whole Blood
ENSG00000213246.6	SUPT4H1	1.00E-08	-0.12	Skin-Sun Exposed (Lower leg)
ENSG00000121101.15	TEX14	3.90E-07	0.28	Thyroid
ENSG00000005379.15	TSPOAP1	0.0000062	0.13	Cells - Cultured fibroblasts
ENSG00000265148.5	TSPOAP1-AS1	0.000091	-0.11	Whole Blood
eQTLs of rs6790 in multi-tissue				
ENSG00000270084.1	GAS5-AS1	2.6e-8	-1.00	Colon - Transverse
ENSG00000152061.17	RABGAP1L	7.2e-7	0.43	Adipose - Subcutaneous
ENSG00000183831.6	ANKRD45	0.0000042	0.56	Cells- Transformed fibroblasts
ENSG00000152061.17	RABGAP1L	0.0000095	0.32	Skin-Sun Exposed (Lower leg)
ENSG00000270084.1	GAS5-AS1	0.000014	-0.38	Cells- Transformed fibroblasts
ENSG00000270084.1	GAS5-AS1	0.000055	-0.84	Adrenal Gland
ENSG00000152061.17	RABGAP1L	0.000065	0.37	Cells- Transformed fibroblasts
